# Thyroid Cancer: Molecular and Modern Advances

**DOI:** 10.1155/2010/317034

**Published:** 2011-02-07

**Authors:** Jennifer E. Rosen, Steven K. Libutti, Stephanie L. Lee

**Affiliations:** ^1^Section of Surgical Oncology and Endocrine Surgery and Boston Medical Center, Boston University School of Medicine, Boston, MA 02118, USA; ^2^Department of Surgery, Montefiore Medical Center, Albert Einstein College of Medicine Yeshiva University, Bronx, NY 10466, USA; ^3^Section of Endocrinology, Diabetes and Nutrition and Boston Medical Center, Department of Medicine, Boston University School of Medicine, Boston, MA 02118, USA

Thyroid cancer is the most common endocrine malignancy and accounts for approximately 44,670 cases and 1,690 deaths in 2010 per the ACS. The purpose of this special issue is to provide an update on recent advances in the understanding of thyroid tumorigenesis and their implications in clinical practice and new surgical approaches to thyroid cancer and to the adoption of techniques used successfully in other tumor types for use in patients with thyroid cancer. Invited papers addressed several of the above topics.

Advances in surgery promise to allow expanded surgical treatment options and potentially make thyroid cancer surgery safer and better accepted by patients. Lang et al. provide a detailed overview of refinement in surgical techniques including endoscopic thyroidectomy techniques, the addition of the da Vinci robot, and the use of operative adjuncts in thyroid surgery such as intraoperative neuromonitoring and quick intraoperative parathyroid hormone. They also outline the clinical studies needed to better analyze the use of this new technology and its relative benefits and risks. Lombardi et al. present their review of a large series of patients with papillary thyroid cancer who underwent video-assisted thyroidectomy (VAT) for completeness of the surgical resection and short-to-medium term recurrence. Their results, while retrospective, seem to indicate that VAT is feasible and safe and may be a valid alternative to conventional surgery for small PTC. No surgical approach is without its concomitant risks—in thyroid surgery, this includes injury to one or both recurrent laryngeal nerves which can result in poor voice quality and the potential for recurrent aspiration. Sanuki et al. discuss their findings of immediate reconstruction of the recurrent laryngeal nerve during thyroid cancer surgery in terms of voice outcomes using videostroboscopic, aerodynamic, and perceptual analyses. While their numbers were small, it addresses an important question often raised in surgery as to whether immediate or delayed reconstruction should be performed.

Advances in genomics have offered exciting insights into the biology of thyroid cancer. While much attention has been focused on the traditional nucleic acids, the study of proteins and their function has been relatively neglected, in part due to the difficulty of the techniques required. The paper by Miyoshi et al. provides a comprehensive overview of the vital role of glycosylation and functional glycomics in thyroid cancer. 

As clinicians well know, an accurate cytological diagnosis is key to the treatment approach for patients with thyroid nodules; this diagnosis is based on distinctive cytological features in combination with immunocytochemistry. Pazaitou-Panayiotou and colleagues present here their study of 83 thyroid cancer fine needle aspirations using Thin Layer Cytology. They present data on a panel of immunomarkers (including Cytokeratin-19, Galectin-3, HBME1, CD-44, CD-56, and E-Cadherin) and describe this promising new technique to improve diagnostic accuracy. 

Early detection is a key component of familial thyroid cancer; Bonora et al. provide us with an excellent review of the familial syndromes, genetic abnormalities, and risk factors proposed to increase the likelihood of this type of neoplasia. Giovanella et al. present a very interesting case example of a rare functioning trabecular tumor of the thyroid gland which serves as a reminder to us that we must not overlook the potential for malignancy in the high-uptake pertechnetate positive nodule.

While the most extensively studied pathway for targeted therapy in thyroid cancer is RAS/RAF/MEK, clinical trials targeting therapies in this pathway are relatively new. The review by Poon and Tai provides us with a comprehensive update of known genomic changes in thyroid cancer and how this information is being used in clinical trials to improve targeted therapy. 

Treatment of certain subtypes of thyroid cancer has been limited in part due to a dearth in therapeutic strategies. In a seminal paper, Dr. Steven Rosenberg proposed the concept that the body's immune system could be manipulated for cancer therapy. (Rosenberg SA (Jan 1984). “Adoptive immunotherapy of cancer: accomplishments and prospects”. Cancer Treat Rep 68 (12): 233–55). Since then, a wide variety of immunotherapeutic approaches have been tested in clinical trials, in particular using either cell-based therapy or immunomodulators. These have led to fairly modest improvements in patient outcomes and a number of therapeutic challenges. This initial work has set the basis for a vigorous research effort to better understand the complex relationship and numerous intersecting pathways which regulate the body's response not only to cancer but to therapy for cancer as well. In their excellent review of dendritic cell-based immunotherapy for the subset of advanced, metastatic, or undifferentiated and anaplastic thyroid carcinoma, Papewalis et al. provide us with a focus on understanding the advances of antitumor immune response in thyroid carcinoma and well summarizes the rationale for adoptive dendritic cell transfer including the technical approach to this interesting new modality of treatment. 

We hope that this special issue will educate, stimulate, and inspire support for participation in clinical trials and new research in the field of thyroid cancer. The development of new surgical approaches, effective targeted therapies, and better preventive interventions will assist in improving treatment and outcome in patients with thyroid cancer. 

*Jennifer E. Rosen* *Jennifer E. Rosen* *Steven K. Libutti* *Steven K. Libutti* *Stephanie L. Lee* *Stephanie L. Lee*

